# Understanding the patient population and test utilization for hepatitis B virus testing

**DOI:** 10.1002/jcla.22987

**Published:** 2019-09-30

**Authors:** Rihwa Choi, Yejin Oh, Seungman Park, Sang Gon Lee, Eun Hee Lee

**Affiliations:** ^1^ Department of Laboratory Medicine Green Cross Laboratories Gyeonggi‐do Korea; ^2^ Department of Laboratory Medicine and Genetics Samsung Medical Center, Sungkyunkwan University School of Medicine Seoul Korea

**Keywords:** hepatitis B virus, Korea, test utilization

## Abstract

**Background:**

Hepatitis B virus (HBV) infection remains a global concern with different epidemiologies due to several factors including migration, vaccination policies, and new antiviral treatment regimens. It is important to understand the characteristics of a patient population, including the prevalence of diseases, and to assess test utilization to understand and evaluate the clinical performance of laboratory tests and to improve the quality of clinical laboratories.

**Materials and methods:**

In this study, we evaluated serologic and virologic laboratory tests including hepatitis B surface antigen, hepatitis B surface antibody, hepatitis B envelope antigen (HBeAg), hepatitis B envelope antibody, and HBV DNA in Korean adults who were exposed to HBV.

**Results:**

During the 1‐year study period, we obtained 22 750 specimens from 17 523 adult Korean patients (>18.0 years; 9894 males and 7629 females) with a median age of 50.1 years (interquartile range, 42.2‐58.2 years). Among them, five serologic and virologic laboratory tests were performed for 1340 (5.9%) specimens from 1172 adult Korean patients (>18.0 years; 647 males and 525 females) with a median age of 46.8 years (range, 19.0‐84.5 years). The prevalence of serologic and virologic tests indicating several clinical situations was evaluated. The correlation coefficient between HBV DNA and HBeAg was *ρ* = 0.85 (*P* < .0001). However, 51.9% (695/1340) of samples did not show agreement between the two test results.

**Conclusions:**

Analysis of the prevalence of patients categorized into five serologic and virologic laboratory results would be helpful to expand our knowledge about patient population characteristics and to improve test utilization.

## 1 INTRODUCTION

Hepatitis B virus (HBV) infection remains a global concern with different epidemiologies due to several factors including migration, vaccination policies, and new antiviral treatment regimens.^1^ In Korea, the prevalence of HBV carriers has changed from 8‐10% in the 1980s and early 1990s to 2.9% in 2013.^2^ Multiple serologic and virologic tests for HBV infection are usually performed simultaneously.^1^, ^3^ Hepatitis B surface antigen (HBsAg) is an envelope protein of HBV detectable in the blood in HBV infection.^3^ HBV surface antibody (anti‐HBs) is an antibody to HBsAg and develops during recovery from hepatitis B and in response to hepatitis B vaccination indicating past infection and immunity.^3^ Hepatitis B envelope antigen (HBeAg) is a marker of viral replication indicating a highly infectious virus.^1^ HBV envelope antibody (anti‐HBe) is an antibody to HBeAg detectable in persons with lower levels of HBV replication.^3^ HBV DNA can be detected and quantified in serum.^1^ Combinations of different positive and negative results of these various tests are used for diagnosis, monitoring of HBV infection, and evaluating treatment responses.^1^, ^4^ Physicians can have difficulties in interpreting laboratory results with different combination of positives and negatives when they do not understand the patient population and their result patterns.^3^, ^5^ It is important to understand the characteristics of a patient population, including the prevalence of diseases, to understand and evaluate the clinical performance of laboratory tests, and to improve the quality of clinical laboratories.^5^

Meanwhile, in the past decade, there has been a lot of studies for the use of serum HBsAg quantification to assess disease activity and monitor treatment response in chronic hepatitis B.^1^, ^6^ Currently, HBsAg test is widely recommended due to the development of new therapies to achieve HBsAg seroclearance indicating functional cure of hepatitis B.^2^ To improve patient outcomes, laboratory tests must be ordered and interpreted appropriately.^7^ Although there have been several studies of serologic and virologic laboratory analyses for HBV, they usually focused on HBsAg seroprevalence, treatment responses, or patient risk for hepatocellular carcinoma.^6^, ^8^ Few studies have involved comprehensive analysis of laboratory results for HBV test utilization. Green Cross Laboratories is one of the largest referral clinical laboratories in Korea, and they administer various laboratory tests for hepatitis B in 514 private clinics nationwide.

Therefore, the aim of this study was to provide information about test result prevalence for different combinations of HBV tests to help physicians and clinical laboratory professionals understand patient population characteristics in Korea by evaluating serologic and virologic laboratory test results of HBsAg, anti‐HBs, HBeAg, anti‐HBe, and HBV DNA in Korean adults exposed to HBV. Furthermore, we investigated the test utilization of those HBV tests in our laboratory, one of the largest referral clinical laboratories in South Korea.

## 2 MATERIALS AND METHODS

### 2.1 Study populations

Data for laboratory test results were obtained through the laboratory information system of Green Cross Laboratories. We obtained test results from adult Korean patients (>18.0 years) with hepatitis B infection who visited private clinics and underwent serum anti‐HBe, HBeAg, and HBV DNA real‐time PCR tests by Green Cross Laboratories between January 2017 and December 2017. Because Green Cross Laboratories is a referral laboratory with limited access to clinical information, samples that were simultaneously tested with for serum anti‐HBe, HBeAg, and HBV DNA real‐time PCR tests were regarded as originating from individuals who were exposed to HBV.^1^, ^4^, ^9^, ^10^ The laboratory results for HBsAg and anti‐HBs tests of those samples were also reviewed. All data were anonymized before analysis. This study was conducted according to the guidelines laid down in the Declaration of Helsinki, and all procedures involving human subjects were approved by the Institutional Review Board of Green Cross Laboratories (GCL‐2018‐1007‐03).

### 2.2 Analytical procedures

Serum HBsAg, anti‐HBs, HBeAg, anti‐HBe tests were performed using a chemiluminescence microparticle immunoassay (CMIA, Abbott) using Architect i2000 (Abbott) according to the manufacturer's instructions. The definition of a positive result in the CMIA test for serum anti‐HBs level included antibody titer ≧10 mIU/mL. For HBsAg, HBeAg, and anti‐HBe tests, the resulting chemiluminescent reaction was measured as relative light units (RLU), and the definition of a positive result for serum HBsAg and HBeAg was ≧1.00 signal‐to‐cutoff (S/CO) ratio (sample RLU/cutoff RLU). The analytical sensitivity for serum HBsAg test ranged from 0.017 to 0.022 IU/mL. For anti‐HBe level, the definition of a positive result was <1.00 S/CO. HBV DNA quantitation tests were performed using COBAS AmpliPrep/COBAS TaqMan HBV Test kits, version 2.0 (Roche) with a sensitivity of 20 IU/mL (116 copies/mL), and the definition of a positive result for HBV DNA was >20 IU/mL. The upper limit of quantitation was 170 000 000 IU/mL (>989 400 000) copies/mL. Categorical variables (positive and negative) are presented as frequencies and percentages.

### 2.3 Statistical analysis

Data normality was assessed by the Shapiro‐Wilks test. Spearman's correlations were calculated to evaluate the relationships between HBV DNA level and HBeAg S/CO values. *P*‐values <.05 were considered to be significant. Statistical analysis was executed using MedCalc Statistical Software version 19.0.3 (MedCalc Software bvba; https://www.medcalc.org; 2019).

## 3 RESULTS

During the 1‐year study period, we obtained 22 750 specimens from 17 523 adult Korean patients (>18.0 years; 9894 males and 7629 females) with a median age of 50.1 years (interquartile range, 42.2‐58.2 years). In 22 750 serum specimens, all three anti‐HBe, HBeAg, and HBV DNA real‐time PCR tests were performed. Among 22 750 specimens that were submitted for follow‐up tests after HBV exposure, the anti‐HBs test was simultaneously requested for 1380 (6.1%) specimens, and the HBsAg test was requested for 1485 (6.5%) specimens. Among 22 750 specimens, 1340 (5.9%) samples from 1172 individuals (647 men and 525 women) with a median age of 46.8 (range, 19.0‐84.5 years) were tested with for HBeAg, anti‐HBe, HBV DNA, HBsAg, anti‐HBs. The laboratory results of 1340 samples for HBV are summarized in Table 1.

**Table 1:** Laboratory results of 1340 samples for hepatitis B virus work‐up and possible interpretation

	HBsAg−/anti‐HBs− (n = 75)	HBsAg−/anti‐HBs+ (n = 68)	HBsAg+/anti‐HBs− (n = 1169)	HBsAg+/anti‐HBs+ (n = 28)^a^	Total (n = 1340)
HBeAg−/anti‐HBe−/HBV DNA−	21^b^	25^c^	57^d^	2^e^	105 (7.8%)
HBeAg−/anti‐HBe+/HBV DNA−	53^f^	42^g^	234^h^	8^i^	337 (25.1%)
HBeAg−/anti‐HBe−/HBV DNA+	0	0	13^j^	0	13 (1.0%)
HBeAg+/anti‐HBe−/HBV DNA−	0	0	88^k^	0	88 (6.6%)
HBeAg+/anti‐HBe+/HBV DNA−	0	0	7^l^	0	7 (0.5%)
HBeAg+/anti‐HBe+/HBV DNA+	0	0	10^m^	0	10 (0.7%)
HBeAg−/anti‐HBe+/HBV DNA+	1^n^	1^o^	577^p^	8^q^	587 (43.8%)
HBeAg+/anti‐HBe−/HBV DNA+	0	0	183^p^	10^r^	193 (14.4%)

a Co‐expression of HBsAg and anti‐HBs occurred in 2.1% of 1340 samples from individuals with HBV exposure.

b Exposure to HBV is uncertain, or HBeAg seroconversion and HBsAg seroconversion are still in progress.

c Results indicating immunity after vaccination or early recovery after HBV infection.

d Results indicating the early phase after HBV infection with acute HBeAg seroconversion in progress, a hepatitis B carrier with undetectable serum HBV DNA on PCR, or a patient under antiviral therapy.

e Results indicating the early phase after HBV infection, a hepatitis B carrier with undetectable serum HBV DNA on PCR, mutations in the S gene region of HBV, or false‐positive results.

f HBeAg seroconversion and HBsAg seroclearance occurred in 4.0% of 1340 samples.

g Recovery and immunity from HBV infection occurred in 3.1% of 1340 samples.

h Results indicating a hepatitis B carrier with undetectable serum HBV DNA on PCR or a patient under antiviral therapy.

i Results indicating HBeAg seroconversion and HBsAg and anti‐HBs co‐expression, a hepatitis B carrier with undetectable serum HBV DNA on PCR, mutations in the S gene region of hepatitis B virus, or false‐positive results.

j Results indicating the early phase of HBV infection or a patient under antiviral therapy.

k Results indicating that the patient might be under antiviral therapy, a hepatitis B carrier with undetectable serum HBV DNA on PCR, or collection of the sample was incorrect.

l Results indicating that the patient might be under antiviral therapy due to HBeAg seroreversion (reappearance of HBeAg in a person who was previously HBeAg‐negative and anti‐HBe‐positive).

m Results indicating that the convalescent window after HBV infection (recovery and HBsAg seroconversion in progress), a patient under antiviral therapy, HBeAg seroreversion (reappearance of HBeAg in a person who was previously HBeAg‐negative and anti‐HBe‐positive), or false‐positive results.

n Results indicating HBV reactivation due to escape mutants, a mutation in the major antigenic determinant of HBsAg, or false‐positive results.

o Results indicating a patient under antiviral therapy, HBV reactivation after HBsAg seroconversion due to escape mutants, a mutation in the major antigenic determinant of HBsAg, or false‐positive results.

p Results indicating a patient under antiviral therapy.

q Results indicating a patient under antiviral therapy, HBeAg seroconversion with detectable HBV DNA, and HBsAg and anti‐HBs co‐expression due to mutations in the S gene region of HBV, or false‐positive results.

r Results indicating a patient under antiviral therapy, HBsAg and anti‐HBs co‐expression due to mutations in the S gene region of HBV, or false‐positive results.

**Figure 1:**
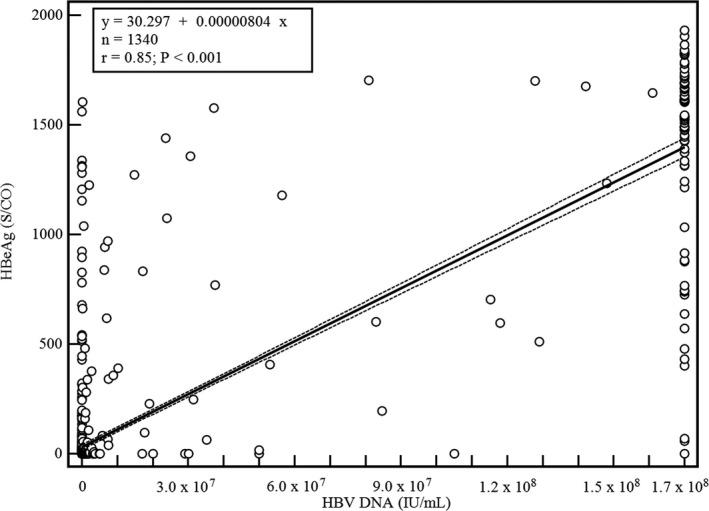
Correlations between HBV DNA level (IU/mL) and HBeAg S/CO values. The black line represents the regression line, and dashed lines are 95% confidence intervals of the regression. Quantification range for HBV DNA test was 20 IU/mL (116 copies/mL) ‐ 170 000 000 IU/mL (989 400 000 copies/mL)

Correlations between HBV DNA level and HBeAg S/CO values were evaluated (Figure 1). The HBV DNA level was significantly associated with HBeAg S/CO values: *ρ *= 0.85 (95% CI 0.84‐0.87) and *P* < .0001, respectively. However, among 537 specimens with HBV DNA level below the quantitation limit (<20.0 IU/mL), 95 (17.7%) showed positive HBeAg (median S/CO 11.8, interquartile range 2.7‐53.8). Among 1042 specimens with negative HBeAg results, 600 (57.6%) had positive HBV DNA results.

## 4 DISCUSSION

In this study, we evaluated 22 750 specimens from 17 523 adults regarded as having chronic HBV infection during the study period. Among them, 1340 (5.9%) specimens were simultaneously tested for HBsAg and anti‐HBs. We evaluated results of 1340 specimens from 1172 adult Korean patients who visited private clinics and requested various HBV serologic tests and provided possible interpretation according to test result combination. Based on existing published guidelines, thresholds for intermediate or high seroprevalence are ≥2% or ≥5% HBsAg, respectively.^3^ The prevalence of HBV carriers in South Korea was reported at 2.9% in 2013.^2^ Because HBV is one of the viruses causing transfusion‐transmitted infection, general population screening and comprehensive monitoring with appropriate HBV tests and interpretation and treatment in HBV patients are important in South Korea.^10^

HBV blood test results can be challenging for physicians to interpret because the complex biological and clinical implications of various HBV tests depend on patient immune response.^11^, ^12^, ^13^ Atypical serologic profiles have puzzled virologists and clinicians since the late 1970s.^12^ We investigate the patient population of various HBV test results and corresponding interpretations based on individual test item results as a table for help in this study.

Although Green Cross Laboratories was limited in terms of clinical information, evaluation of requested test items could be used to make assumptions about the patient population based on the knowledge that the HBeAg, anti‐HBe, and HBV DNA test were performed for patients exposed to HBV in Korea.^4^ Although HBV DNA level and HBeAg S/CO values were significantly associated, their results were discrepant for more than half the specimens (695/1,340, 51.9%). Because HBeAg is not essential for viral replication and the HBeAg loss or seroclearance rate varies according to viral DNA replication, both tests are recommended to be performed simultaneously to monitor chronic HBV infection.^1^, ^3^, ^10^, ^14^

According to recommendations from the 2015 and 2018 Korean Association for Study of the Liver guidelines for management of chronic hepatitis B, 2017 European Association for the Study of the Liver clinical practice guidelines on the management of HBV infection, and 2016 American Association for the Study of Liver Diseases guidelines for treatment of chronic hepatitis B, the HBsAg test should be used to achieve HBsAg seroclearance.^1^, ^4^, ^9^, ^10^ Although the gold standard measurement of treatment response to new antiviral treatments is HBsAg seroclearance as a functional cure of hepatitis B, HBsAg was not tested simultaneously in 93.5% of samples (21 265/22 750).^7^ Considering that test utilization management might improve health outcomes, 93.5% of samples that were not previously analyzed by HBsAg test might have been more appropriately evaluated for the prevalence based on serologic and virologic laboratory results.^12^ In South Korea, the HBsAg quantification test has been covered by national insurance in chronic hepatitis patients since January 2018. The Korean Association for Study of the Liver revised the guidelines for chronic hepatitis B clinical practice in November 2018.^10^ Reimbursement might be an important factor for test utilization.^7^ Additional studies about test utilization and clinical impact of HBsAg quantification tests in Korean chronic HBV patients are needed to improve patient outcomes.^7^

In this study, 4.0% (53/1340) of samples exhibited HBsAg seroclearance and 3.1% (42/1340) of samples exhibited HBsAg seroconversion under treatment (Table 1). This was comparable with previous studies on HBsAg seroclearance.^1^, ^7^, ^15^ Various rates of HBsAg seroclearance have been reported among different populations under different treatment regimens.^1^, ^7^ The results indicated that co‐expression of HBsAg and anti‐HBs occurred in 2.1% (28/1340) of this study population. Co‐expression of HBsAg and anti‐HBs has been hypothesized to result from primary infection with multiple subtypes followed by clearance of one, from viral strains carrying HBsAg mutations that escape anti‐HBs neutralization, or from change in the primary sequence of the ‘‘a’’ determinant in the S gene region of HBV that alters its antigenic structure.^12^ However, the clinical significance of many mutations on HBsAg antigenicity still remains to be clarified.^6^, ^7^, ^12^ Because the clinical significance of patients with those test results should be further studied and improved in accordance with various treatment regimens, it is important to know about the prevalence change.^12^, ^16^

The limitation of this study was the lack of clinical information including detailed history and physical examination and other laboratory and image studies associated with HBV infection and disease severity. However, because the test indications and clinical management guidelines for HBV infection can provide valuable hints about the clinical situation and monitoring test utilization is an important part of quality improvements of clinical laboratory, this study provides a valuable example for clinical laboratories with limited clinical information. Future studies about long‐term change in the prevalence in the patient population are needed.

In conclusion, we investigated serologic and virologic laboratory HBV test results from private clinics nationwide in Korea. Evaluation of the prevalence in the laboratory test results can help to expand our knowledge about patient population characteristics and improve test utilization.

## CONFLICT OF INTEREST

None.

## AUTHOR CONTRIBUTIONS

RC designed the study. RC, YO, SP, and SG were responsible for data acquisition. R.C and YO analyzed and interpreted the data. RC wrote the article. All authors read and approved the final manuscript.

## ETHICAL APPROVAL

This study was conducted according to the guidelines laid down in the Declaration of Helsinki, and all procedures involving human subjects were approved by the Institutional Review Board of Green Cross Laboratories (GCL‐2018‐1007‐03). The Institutional Review Board of Green Cross Laboratories waived informed consent for the retrospective data collection and review.
